# TRPM4 Expression During Postnatal Developmental of Mouse CA1 Pyramidal Neurons

**DOI:** 10.3389/fnana.2021.643287

**Published:** 2021-04-28

**Authors:** Denise Riquelme, Oscar Cerda, Elias Leiva-Salcedo

**Affiliations:** ^1^Departamento de Biología, Facultad de Química y Biología, Universidad de Santiago de Chile, Santiago, Chile; ^2^Programa de Biología Celular y Molecular, ICBM, Facultad de Medicina, Universidad de Chile, Santiago, Chile; ^3^Millennium Nucleus of Ion Channel-Associated Diseases, Santiago, Chile

**Keywords:** TRPM4, ICAN, hippocampus, CA1, pyramidal neurons

## Abstract

TRPM4 is a non-selective cation channel activated by intracellular calcium and permeable to monovalent cations. This channel participates in the control of neuronal firing, neuronal plasticity, and neuronal death. TRPM4 depolarizes dendritic spines and is critical for the induction of NMDA receptor-dependent long-term potentiation in CA1 pyramidal neurons. Despite its functional importance, no subcellular localization or expression during postnatal development has been described in this area. To examine the localization and expression of TRPM4, we performed duplex immunofluorescence and patch-clamp in brain slices at different postnatal ages in C57BL/6J mice. At P0 we found TRPM4 is expressed with a somatic pattern. At P7, P14, and P35, TRPM4 expression extended from the soma to the apical dendrites but was excluded from the axon initial segment. Patch-clamp recordings showed a TRPM4-like current active at the resting membrane potential from P0, which increased throughout the postnatal development. This current was dependent on intracellular Ca^2+^ (I_*CAN*_) and sensitive to 9-phenanthrol (9-Ph). Inhibiting TRPM4 with 9-Ph hyperpolarized the membrane potential at P14 and P35, with no effect in earlier stages. Together, these results show that TRPM4 is expressed in CA1 pyramidal neurons in the soma and apical dendrites and associated with a TRPM4-like current, which depolarizes the neurons. The expression, localization, and function of TRPM4 throughout postnatal development in the CA1 hippocampal may underlie an important mechanism of control of membrane potential and action potential firing during critical periods of neuronal development, particularly during the establishment of circuits.

## Introduction

TRPM4 is a Ca^2+^ -activated non-selective cation channel (CAN) permeable to monovalent cations. TRPM4 and TRPM5 are the only members of the TRP family directly activated by intracellular Ca^2+^ (Launay et al., 2002; Prawitt et al., 2003; Nilius et al., 2005; Pedersen et al., 2005). TRPM4 is widely expressed in several tissues, including the brain (Shpak et al., 2012; Funk, 2013; Kim et al., 2013; Lei et al., 2014; Riquelme et al., 2018). In neurons, TRPM4 is gated by intracellular Ca^2+^ increases through the activation of neurotransmitter receptors such as G_*q*_ -coupled receptors or ionotropic receptors (Mrejeru et al., 2011; Menigoz et al., 2016). TRPM4 has been proposed as the I_*CAN*_ underlying the plateau potential in accessory olfactory neurons (Shpak et al., 2012) and hypothalamus (Teruyama et al., 2011), and it has been confirmed as the I_*CAN*_ in pre-Bötzinger neurons, pyramidal neurons in the hippocampus CA1 (Menigoz et al., 2016), layer IX cerebellar Purkinje neurons (Kim et al., 2013), and in the neurons of the thalamic reticular nuclei (O’Malley et al., 2020).

TRPM4 may help to control both the membrane potential and the firing frequency of neurons. In cerebellar Purkinje neurons and thalamic reticular nucleus neurons, activation of mGluR1 increases the intracellular Ca^2+^, activating TRPM4, which then increases the firing frequency through an afterdepolarization or a plateau potential, respectively, (Shin et al., 2009; Kim et al., 2013; O’Malley et al., 2020). Furthermore, the activation of mGluR5 increases TRPM4 activity in pre-Bötzinger neurons, thus controlling the motor output of the respiratory central pattern generator (Mironov, 2008; Picardo et al., 2019). These antecedents suggest TRPM4 may play a significant role in the control of neuronal excitability and firing frequency more generally.

In the hippocampal area CA1, the activation of NMDA receptors increases postsynaptic calcium levels, triggering the activity of TRPM4, which in turn increases postsynaptic depolarization, thus enhancing long-term potentiation (LTP) of excitatory transmission (Menigoz et al., 2016). Moreover, TRPM4 knockout mice show a variety of deficits in hippocampus-related behavioral tasks linked to decreased LTP (Bovet-Carmona et al., 2018, 2019). TRPM4 is also involved in neuronal degeneration and ischemia/reperfusion damage (Schattling et al., 2012; Leiva-Salcedo et al., 2017), where it plays a key role in oncotic cell swelling and neuronal death, leading to the proposal that TRPM4 inhibition could be a treatment for these conditions (Jha et al., 2020; Luo et al., 2020; Robert et al., 2020).

Although TRPM4 expression in CA1 pyramidal neurons has been addressed through immunoblot, *in situ* hybridization, mRNA expression, and cell immunofluorescence, no information about its subcellular localization and expression during postnatal development has been reported. Here, we addressed the localization of TRPM4 in CA1 and its functional expression in pyramidal neurons during postnatal development. We found TRPM4 is localized in the soma and apical dendrites of pyramidal neurons in the *stratum pyramidale*, with only sparse expression in the *stratum oriens*. Furthermore, we found the presence of a Ca^2+^ -activated non-selective cation current (I_*CAN*_), sensitive to 9-Phenanthrol (a TRPM4 inhibitor), which increases during postnatal development and is active at the resting membrane potential. These data support the hypothesis that TRPM4 plays multiple roles in controlling neuronal excitability and suggest important roles in pathological conditions.

## Methods

### Animals and Tissue Sectioning

All experiments were conducted following animal protocols approved by the Ethical Committee of the Universidad de Santiago de Chile, according to the rules and guidelines of the National Agency of Research and Development (ANID). Male and female C57BL/6J mice were housed in a temperature and humidity-controlled facility with a 12/12 h light/dark cycle with water and food *ad libitum*. Male and female mice at postnatal day P0 and male mice at P7, P14, and P35 were used for immunofluorescence and electrophysiology experiments. All mice were obtained from independent litters.

Coronal brain slices containing the dorsal hippocampus were prepared from mice between P0 to P35 (Supplementary Figure 1). Briefly, male mice at P7, P14, and P35 were deeply anesthetized by isoflurane inhalation (3%). Animals at P35 were intracardially perfused with PBS 0.1 M pH 7.2 followed by freshly made 4% w/v formaldehyde dissolved in PBS 0.1 M pH 7.4. All mice were euthanized by decapitation, and brains were quickly removed and placed in 4% w/v formaldehyde (Merck, Germany), then incubated overnight at 4°C. The next day, the brains were placed in a vibrating tissue slicer (1000 plus, Vibratome, United States), and sectioned using a sapphire blade to get 60 μm thick slices.

### Primary Antibodies

Monoclonal anti-TRPM4 L88/86 antibody was obtained from hybridoma tissue cultures and used as non-diluted supernatant (RRID: AB_2716758). The antibody was validated by immunoblots in HEK293 expressing TRPM4, by immunofluorescence in B16-F10 cells expressing an shRNA against TRPM4 (Riquelme et al., 2018), and in cerebellar lobule XI (positive labeling) and lobule IV (negative labeling; Supplementary Figure 2). Polyclonal anti-MAP2 antibody (ab32454, RRID: AB_776174) was obtained from Abcam (United States). Anti-AnkG antibody (75-146, RRID: AB_10673030) was obtained from NeuroMab (United States; Supplementary Table 1).

### Secondary Antibodies

Alexa Fluor 546 goat anti-mouse (A21133), Alexa Fluor 546 donkey anti-rabbit (A10040), and Alexa Fluor 488 goat anti-mouse IgM (A21042) antibodies were obtained from Life Technologies (United States; Supplementary Table 1).

### Immunofluorescence Labeling

Tissue sections containing the hippocampus were blocked in 10% v/v normal goat serum (Sigma, United States) in 0.1 M PBS, pH 7.4 (NGS) for 1 h at room temperature (RT); then sections were incubated with the primary antibody used as non-diluted supernatant or diluted in the same NGS solution using gentle rocking for 60 h at 4°C. Sections were washed in 0.1 M PBS for 30 min before incubation with the secondary antibody (1.5 h at RT). After 6 washes of 5 min with PBS, the sections were mounted on glass slides using Mowiol mounting media and covered with a 0.17 mm thick coverslip. Fluorescence was observed in an LSM-510 or LSM 800 inverted confocal microscope (Zeiss, Germany). For full area composition, images were acquired with a 10x, 0.3 N.A. objective. High magnification confocal images were acquired using a 40 × 1.4 N.A. oil immersion objective, with a pinhole of 1 Airy unit for each channel, a scan zoom of 1, frame scan mode with averaging of 4, and a pixel time of 2.06 μs. All images were obtained at 8 or 16-bit pixel depth at 1024 × 1024 resolution. Images were adjusted for overall brightness, contrast, and level using Fiji-ImageJ. For intensity measurements, confocal images were acquired using a 25 × 0.8 N.A. water immersion objective, with a pinhole of 1 Airy unit for each channel, a scan zoom of 0.5, frame scan mode with an averaging of 4, and a pixel time of 0.52 μs. Images were taken as *Z*-stack of 3 images at 1.5 μm intervals and analyzed as *Z*-projections using Fiji-ImageJ.

### Image Analysis

Intensity measurements were collected using Fiji-ImageJ with the regions of interest (ROI) plugin. Labeling intensity throughout the *stratum pyramidale*, *stratum radiatum*, and *stratum oriens* of hippocampal area CA1 was measured using several rectangular ROI of 31 × 52 μm. Data were collected for at least four animals on each postnatal day and the fluorescence intensity was normalized to the fluorescence of the *stratum pyramidale* of each mouse. For image analysis statistics, we tested for normality using Kolmogorov-Smirnov and the statistical significance was determined by the Kruskal–Wallis test followed by Dunn’s *post hoc* test.

### Electrophysiological Recordings

Mice (C57BL/6J) between postnatal day 7-35 were deeply anesthetized with 3% isoflurane (P0 mice were not anesthetized) and their brains were quickly removed and placed in ice-cold oxygenated (95% O_2_, 5% CO_2_) high-magnesium ACSF containing (in mM): 124 NaCl, 2.5 KCl, 5 MgCl_2_, 0.5 CaCl_2_, 1.25 NaH_2_PO_4_, 0.4 ascorbic acid, 2 sodium pyruvate, 26 NaHCO_3_, 11 glucose, and pH 7.4. Tissue blocks containing the hippocampus were placed in a vibratome to obtain parasagittal brain slices (350 μm thick). Then, slices were transferred to a chamber containing oxygenated ACSF containing (in mM): 125 NaCl, 2.5 KCl, 1.3 MgCl_2_, 2.5 CaCl_2_, 1.25 NaH_2_PO_4_, 26 NaHCO_3_, 11 glucose, and pH 7.4. After 1.5 h of recovery, the slices were transferred to a recording chamber mounted on a Zeiss Axioscope 200FS DIC microscope. Slices were continuously perfused with oxygenated ACSF (2–3 mL/min) at 32 ± 2°C.

Voltage and current-clamp experiments. Whole-cell recordings were performed from *stratum pyramidale* neurons in the hippocampus using borosilicate glass pipettes (4 to 6 GΩ) filled with intracellular solution. For current-clamp, the intracellular solution contained (in mM): 130 potassium-gluconate, 10 KCl, 10 HEPES, 1.5 EGTA, 2 Mg-ATP, 0.3 Na-GTP, and pH 7.2 adjusted with KOH (∼300 mOsm); and for voltage-clamp intracellular solution contained: 130 CsCh_3_SO_3_, 8 NaCl, 10 HEPES, 5 TEA-Cl, 10 EGTA, 2 Mg-ATP, 0.3 Na-GTP, 5 QX-314, and pH 7.2 adjusted with CsOH (∼300 mOsm/kg). For perforated-patch protocols, 300 μg/mL nystatin diluted in DMSO was added to the intracellular solutions. Usually, a stable access resistance (R_*a*_) was obtained after 10 to 15 min (*R*_*a*_ = 10–20 MΩ). In the case of a sudden decrease in the access resistance, the recording was discarded.

For voltage-clamp recordings, the pipette and whole-cell capacitance, as well as series resistance, was compensated by 80%, neurons were held at -70 mV and recorded in ACSF containing (in μM): 1 TTx, 50 CNQx, 25 DL-AP5, and 100 picrotoxin. Voltage-ramp protocols from -100 to 100 mV (0.4 mV/ms) from a holding potential of -70 mV were delivered somatically at 0.2 Hz. Voltage-clamp recording was performed using a Multiclamp 700A (Molecular Devices, United States) or HEKA EPC10 (HEKA GmBH, Germany), data were filtered at 10 kHz and digitized at 20 kHz using pClamp 10.3 or HEKA Patchmaster 2 × 73.5.

### Data Analysis

Electrophysiological data were analyzed using Clampfit 10.3. Data are reported as mean ± standard deviation in the text and a 95% confidence interval (CI) in the plots. Data were tested for normal distribution using the Kolmogorov–Smirnov test. For parametric data, statistical significance between groups means was assessed using one-way ANOVA followed by a Dunnett’s multiple comparisons *post hoc* test. For the two-group comparison, we used the *t*-student test. Statistical significance was determined at *p* < 0.05. The effect size was calculated using Cohen’s d analysis for shared control using Estimation statistics beta analysis^1^ (Ho et al., 2019).

## Results

### TRPM4 Expression in CA1 Pyramidal Neurons

**FIGURE 1:**
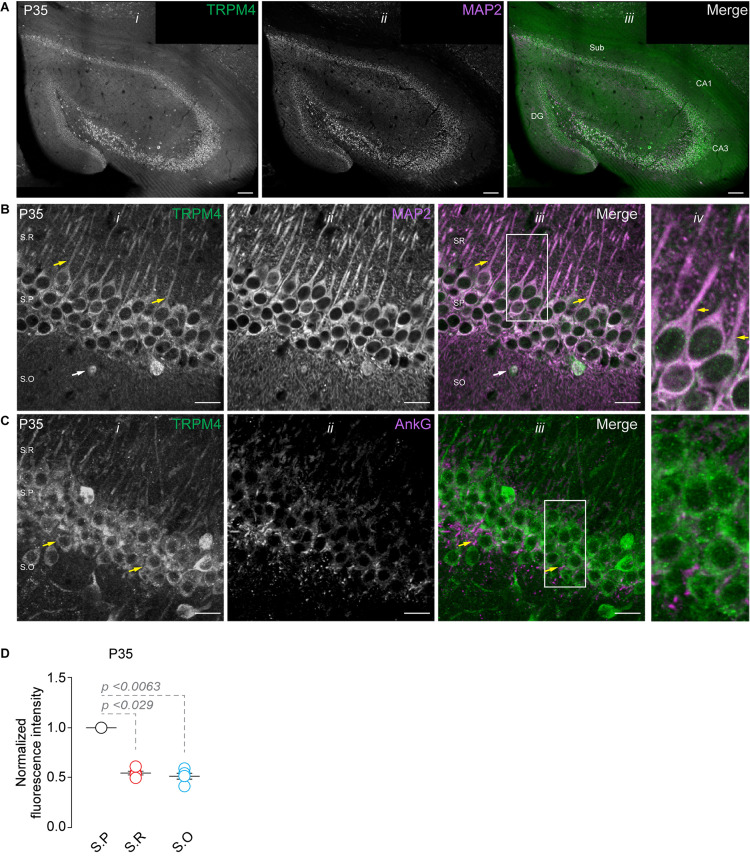
TRPM4 expression in CA1 pyramidal neurons at P35. Confocal images showing double labeling of **(A)** TRPM4 (Alexa 488, green, *i*) with MAP2 (Alexa 546, magenta, *ii*), merged signals (*iii*), in the whole hippocampal area. **(B)** Shows the expression of TRPM4 (*i*) MAP2 (*ii*) and the merged signals (*iii*) in area CA1, and (*iv*) shows a zoomed area (white rectangle); yellow arrowheads show neurons in the *stratum pyramidale* and the distribution in the soma and proximal apical dendrite, white arrowhead show the distribution in the *stratum oriens*, showing the expression in the somatic region of the neurons. **(C)** Shows the expression of TRPM4 (Alexa 488, green, *i*) and AnkG (Alexa 546, magenta, *ii*) and the merged signals (*iii*), and (*iv*) shows a zoomed area (white rectangle). **(D)** Graph of normalized fluorescence intensity of TRPM4 in S.P, S.R, and S.O of area CA1. Fluorescence intensity values were normalized to CA1 S.P for each mouse. Each point corresponds to an individual mouse (Kruskal–Wallis followed by Dunn’s *post hoc* test vs. CA1 S.P; *n* = 5 mice). S.P, *stratum pyramidale*; S.R, *stratum radiatum*; and S.O, *stratum oriens*. Calibration bars = 100 μm in **(A)**, and 20 μm in **(B,C)**.

Analysis of the Allen brain map database shows the presence of TRPM4 mRNA in the pyramidal layer of the hippocampal area CA1 of adult mice, with a pattern suggesting its expression in pyramidal neurons. To characterize the expression of TRPM4 in pyramidal neurons from the hippocampal area CA1, we performed duplex immunofluorescence labeling with TRPM4 and MAP2 and TRPM4 and AnkG in mouse brain sections from P35 animals. As expected, we found strong TRPM4 labeling in the whole hippocampal formation, particularly in the pyramidal layer of CA1, CA3, subiculum, and in the granular cells of the dentate gyrus (Figure 1A). A detailed analysis of the CA1 region shows TRPM4 labeling in the soma and in the apical dendrites of the pyramidal neurons that extend through the *stratum radiatum*; the labeling is present in the proximal and middle apical dendrites but is not present in the dendritic branches (Figure 1B); the axon initial segment (AIS) is devoid of TRPM4 labeling (Figure 1C). In the *stratum oriens*, we found sparse TRPM4 expression restricted in most cases to the somatic region and without labeling of the basal dendrites (Figure 1B). The labeling intensity measurements show higher intensity in the *stratum pyramidale* and a reduction in the *stratum radiatum* and *stratum oriens* (Figure 1D). Thus, TRPM4 is localized in the soma and apical dendrites of pyramidal neurons and is absent in the AIS.

### TRPM4 Expression During Postnatal Development of CA1

**FIGURE 2:**
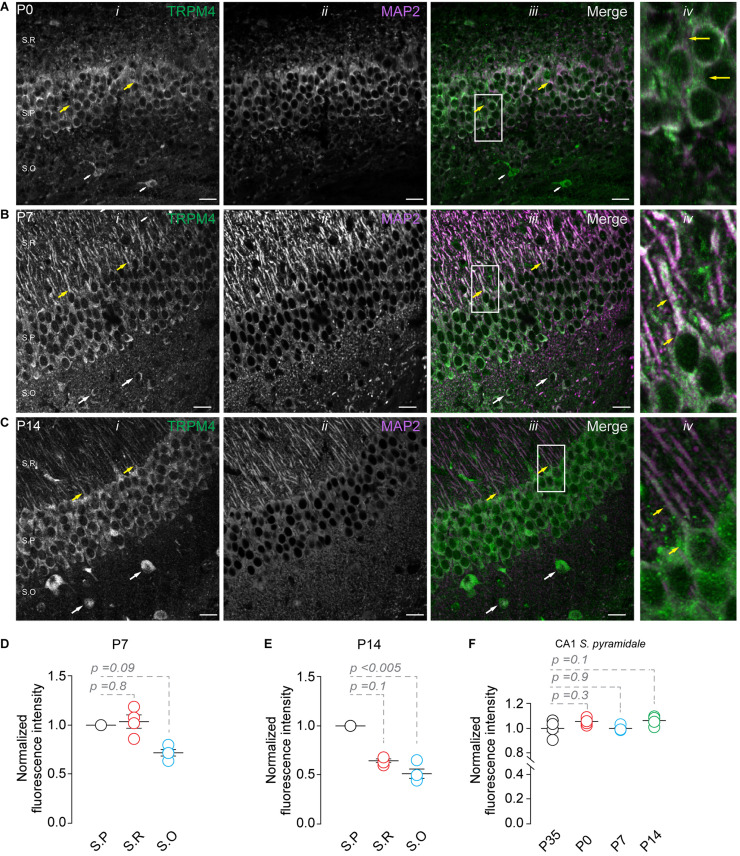
TRPM4 expression in CA1 pyramidal neurons during postnatal development. Confocal images showing double labeling of TRPM4 (Alexa 488, green, *i*) with MAP2 (Alexa 546, magenta, *ii*), the merged signals (*iii*), and (*iv*) shows a zoomed area (white rectangle), yellow arrowheads showed neurons in the *stratum pyramidale* and the distribution in the soma and proximal apical dendrite; white arrowhead shows the distribution in the *stratum oriens*, showing the expression in the somatic region of the neurons. **(A)** Shows the expression of TRPM4 and MAP 2 at P0, **(B)** Shows the expression at P7, and **(C)** Shows the expression at P14. **(D,E)** Graphs of normalized fluorescence intensity of TRPM4 in S.P, S.R, and S.O of area CA1 of P7 and P14. Fluorescence intensity values were normalized to CA1 S.P. for each mouse. Each point corresponds to an individual mouse (Kruskal Wallis followed by Dunn’s *post hoc* test vs. CA1 S.P; *n* = 4 mice). **(F)** Graph of normalized fluorescence intensity of TRPM4 in S.P, from P0, P7, P14, and P35 mice. Fluorescence intensity values were normalized to mean intensity values of CA1 S.P of P35 mice. Each point corresponds to an individual mouse (Kruskal Wallis followed by Dunn’s *post hoc* test vs. CA1 S.P, P35; *n* = 4–5 mice). S.P, *stratum pyramidale*; S.R, *stratum radiatum*; and S.O, *stratum oriens*. Calibration bars = 20 μm.

Next, we studied the expression of TRPM4 at different postnatal stages. We performed duplex immunofluorescence labeling for TRPM4 and MAP2 in brain sections containing CA1 at P0, P7, and P14. At P0, we found a compact and thicker pyramidal layer with a small *stratum radiatum* (Soriano et al., 1994; Supèr and Soriano, 1994; Andersen et al., 2006, 116); TRPM4 is expressed in the soma of the neurons present in the pyramidal layer (Figure 2A, yellow arrows). Moreover, we found a few neurons expressing TRPM4 in the *stratum oriens* (Figure 2A, white arrows). At P7, we found a defined neuronal morphology and a clear separation between the hippocampal layers. Additionally, neurons in the *stratum pyramidale* show a strong somatic and apical dendritic labeling (Figure 2B, yellow arrows), with sparse labeling in the *stratum oriens* (Figure 2B, white arrows). The labeling intensity in the *stratum pyramidale* is similar to the *stratum radiatum* but decreases in the *stratum oriens* (Figure 2D). In P14, we found TRPM4 labeling in the somatic region of the pyramidal layer with a similar pattern as in P35, in which the labeling occurs in *stratum pyramidale* and the *stratum radiatum*. The neurons show labeling at the soma and the main apical shaft (Figure 2C, yellow arrows). TRPM4 is sparsely expressed in neurons present in the *stratum oriens* with a somatic pattern and without labeling of the basal dendrites (Figure 2C, white arrows, and Figure 2E). When we compared the fluorescence intensity of the *stratum pyramidale* between the different postnatal stages, we found the expression in the soma remains unchanged between the different postnatal stages (Figure 2F). Together, our results indicate TRPM4 is expressed at birth, and neurons progress from a somatic pattern to a somatodendritic pattern with labeling in the apical dendrites throughout postnatal development.

### TRPM4 Functional Expression in CA1 Pyramidal Neurons During Postnatal Development

**FIGURE 3:**
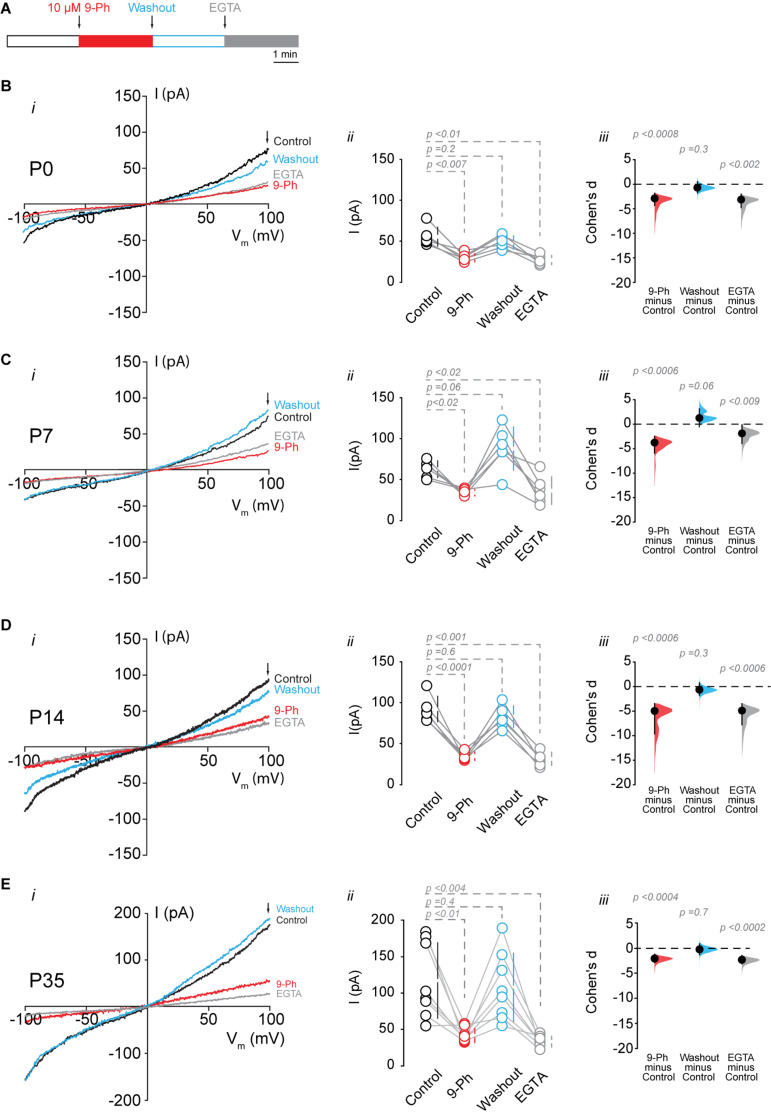
TRPM4 functional expression in CA1 pyramidal neurons during postnatal development. Current recordings in CA1 pyramidal neurons in response to a somatic voltage-ramp (−100 to 100 mV from a holding potential of −70 mV), glutamatergic transmission and action potential generation was inhibited (see section “Methods”). Neurons were recorded in a nystatin-perforated patch configuration (control, 9-Ph and washout) and after breaking the membrane to enter whole cell configuration, which allows EGTA diffusion (EGTA). **(A)** Diagram of the experimental protocol used in the electrophysiological recordings. **(B)** Shows the currents recorded at P0 (*n* = 6), **(C)** shows the currents at P7 (*n* = 6), **(D)** shows the currents at P14 (*n* = 6), and **(E)** shows the currents at P35 (*n* = 8). (*i*) Each representative current trace was obtained from CA1 pyramidal neurons (*stratum pyramidale*) after different treatments (Control, 9-Ph, Washout, EGTA); arrowhead shows where the current was measured. (*ii*) Summary plot of the current in the different treatments, vertical lines show the 95% CI (statistical difference were determined using a one-way ANOVA, *p*-values are show above each group). (*iii*) Cohen’s *d* for 3 comparisons against the shared control. Mean difference is depicted as a dot, the vertical bar shows the 95% CI as indicated by the end of the vertical error bars, and the *p*-values are shown above each point, depicting results of a two-sided permutation *t*-test. Each point corresponds to an individual mouse (1 slice per mouse).

To determine the functional expression of TRPM4, we performed nystatin-perforated patch-clamp recordings in pyramidal cells in CA1 at P0, P7, P14, and P35. We measured the TRPM4 current in pyramidal neurons using glutamatergic and Na^+^ channel blockers [(Riquelme et al., 2018), see section “Methods”]. We found that a somatic voltage ramp (-100 to 100 mV) in pyramidal neurons at P0 activates a non-rectifying current with a maximum of 55.8 ± 11.6 pA, and a *V*_*rev*_ = 0.9 ± 1.3 mV. The application of 10 μM 9-Ph reduces the current to 30.2 ± 4.9 pA. This effect is reversible after ∼3 min washout (48.9 ± 7.9 pA) with no changes in the *V*_*rev*_. To further confirm the CAN nature of this current, we broke the membrane to enter the whole-cell configuration, thus allowing the diffusion of EGTA. This experimental protocol (Figure 3A) reduced the current to 27.4 ± 5.6 pA (Figure 3B). We found comparable results at P7: the current was non-rectifying with a *V*_*rev*_ = 1.4 ± 0.4 mV, and a peak current of 63.2 ± 10.1 pA, the application of 9-Ph reduced the current to 34.2 ± 3.9 pA, and was completely recovered after washout (88.4 ± 26.4 pA). The intracellular diffusion of EGTA reduced the current to 36.8 ± 17.1 pA (Figure 3C), with no change in the *V*_*rev*_.

At P14, we found a non-rectifying current with a *V*_*rev*_ = −1.8 ± 1.9 mV, and a maximal current of 91.4 ± 15.6 pA. This current was reduced to 33.8 ± 5.2 pA after the application of 10 μM 9-Ph. Washout recovered the maximal current to 82.1 ± 13.3 pA, and EGTA diffusion reduced the current to 30.5 ± 8.2 pA (Figure 3D). Similarly, at P35, we found a non-rectifying current with a maximum of 117.4 ± 51.7 pA and a *V*_*rev*_ = −1.2 ± 2.6 mV (Figure 3E, ***i***, black trace, and ***ii*** black circles). The application of 9-Ph reduced the current (42.2 ± 9.7 pA, one-way ANOVA, *p* < 0.01, Figure 3E, ***i*** red trace, and ***ii*** red circles); current levels were recovered after washout (108 ± 46.7 pA, Figure 3E, ***i*** blue trace, and ***ii*** blue circles), and EGTA diffusion reduced the current (33.5 ± 7.3 pA, one way ANOVA, *p* < 0.004, Figure 3E, ***i*** gray trace, and ***ii*** gray circles), with no changes in the *V*_*rev*_. Thus, these results indicate an increase in the 9-Ph sensitive and Ca^2+^ -dependent current during postnatal development.

### Resting Membrane Potential Through the Postnatal Development of CA1 Pyramidal Neurons

**FIGURE 4:**
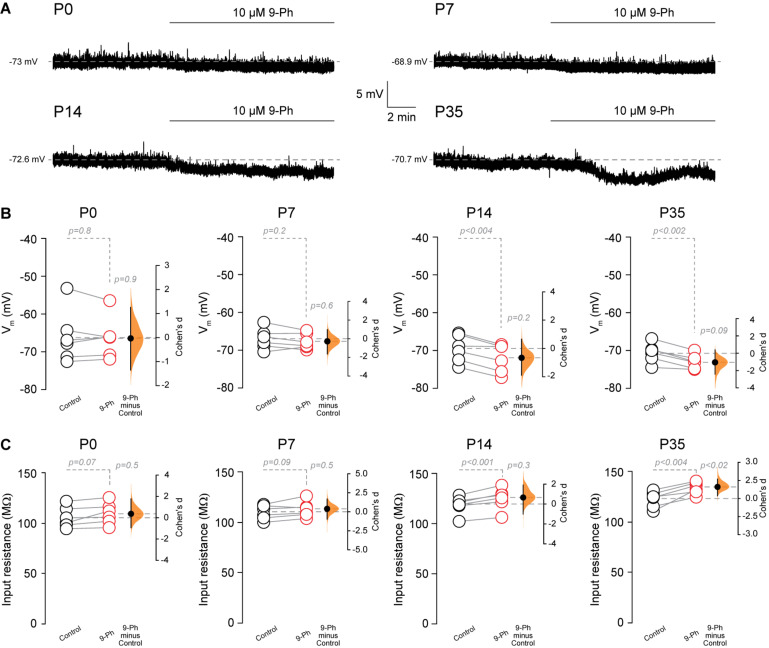
TRPM4 inhibition hyperpolarizes CA1 pyramidal neurons during postnatal development. **(A)** Representative perforated-patch voltage trace recorded in pyramidal neurons showing the effect of 10 μM 9-Ph at P0, P7, P14, and P35. **(B)** Summary plots showing resting membrane potential and the effect of 10 μM 9-Ph at P0 (*n* = 6), P7 (*n* = 6), P14 (*n* = 6), and P35 (*n* = 6). **(C)** Summary plots showing the effect of 9-Ph on the input resistance at P0 (*n* = 6), P7 (*n* = 6), P14 (*n* = 6), and P35 (*n* = 6; statistical difference were determined using a one-way ANOVA, *p*-values are show above each group). On the right side of each plot the paired mean difference between the conditions is shown; the mean difference is depicted as a dot; the 95% confidence interval is indicated by the end of the vertical error bar. Statistical differences were evaluated using paired *t*-test and *p*-values are shown above. Each point corresponds to an individual mouse (1 slice per mouse).

Next, we assessed the effect of TRPM4 inhibition at different postnatal stages. We performed current-clamp experiments using the nystatin-perforated patch-clamp configuration in pyramidal neurons in CA1 from P0, P7, P14, and P35 mice. We found TRPM4 inhibition at P0 and P7 did not change the membrane potential (P0 control = −66.1 ± 7 mV; P0 9-Ph = -66.3 ± 5.5 mV, *t*-test, *p* < 0.8; P7 control = −67 ± 2.7 mV; P7 9-Ph = −67.8 ± 2 mV, *n* = 6, *t*-test, *p* < 0.2). At P14 and P35, we found that 10 μM 9-Ph reversibly reduced the membrane potential (P14 control = −69.6 ± 3.6 mV; P14 9-Ph = −72.1 ± 3.8 mV, *t*-test, *p* < 0.004; P35 control = −70.7 ± 2.6 mV; P35 9-Ph = −73.1 ± 1.9 mV, *n* = 6, *t*-test, *p* < 0.002; Figures 4A,B). These membrane potential changes were associated with an increase in the input resistance (Figure 4C), suggesting the closing of a conductance. Together, these results show that a Ca^2+^ -activated, 9-Ph sensitive conductance participates in controlling the membrane potential at specific stages of postnatal development.

## Discussion

Here we report the distribution of TRPM4 in hippocampal area CA1 during postnatal development in mice. Using a combination of immunofluorescence with double labeling to localize TRPM4-MAP2 or TRPM4-AnkG, and electrophysiology, we found (1) that TRPM4 is expressed at birth with constant expression through postnatal development; (2) TRPM4 expression is restricted to the soma and apical dendrites, and (3) a CAN current sensitive to 9-Ph, which is consistent with TRPM4, is active at resting membrane potential and increases its magnitude through postnatal development.

### TRPM4 Expression in CA1 Pyramidal Neurons During Postnatal Development

In mice, the expression of TRPM4 has been demonstrated by immunoblot, reverse transcription qPCR, and immunofluorescence in hippocampal neuron cultures (Schattling et al., 2012). The expression of TRPM4 in the hippocampal area CA1 has been addressed by *in situ* hybridization and functionally demonstrated in knockout mice, suggesting a role in NMDA-induced postsynaptic depolarization, thus boosting depolarization and enhancing LTP of excitatory transmission (Menigoz et al., 2016; Bovet-Carmona et al., 2018), an effect similar to that observed in dopaminergic neurons in the substantia nigra, where a 9-Ph sensitive current participates in postsynaptic depolarization (Mrejeru et al., 2011), despite this information the expression of TRPM4 at the cellular level during postnatal development has not been thoroughly addressed.

We found TRPM4 is expressed at birth, with little variation in the level of the channel but with marked changes in its localization during postnatal development, adopting a somatodendritic pattern; dendritic expression is restricted to the apical dendritic tree. This change is temporally related to an increase in a Ca^2+^ -dependent and 9-Ph sensitive current throughout development. In this context, it is notable that CA1 pyramidal neurons transition from network-driven burst firing to an intrinsically bursting pattern during development. This transition is the emergence of an Afterdepolarization Potential (ADP) arising from voltage-dependent Ca^2+^ channels and a persistent Na^+^ current that increases the ADP duration, which reaches its maximum at P20 (Wu et al., 2004). However, ADP is not fully explained by this mechanism; we suggest TRPM4 may participate in ADP, as in other neuronal types (Mironov, 2008; Kim et al., 2013; Lei et al., 2014; Picardo et al., 2019). Our hypothesis is supported by evidence that the ADP in CA1 pyramidal neurons is partially dependent on a CAN current (Fraser and MacVicar, 1996).

### Localization of TRPM4 in CA1 Pyramidal Neurons

Localization of the ion channels determines its influence on the membrane potential and its ability to control synaptic transmission and excitability (Trimmer, 2015). The apical dendrites of CA1 pyramidal neurons receive input primarily from distant CA3 neurons through the Schaffer collaterals, while distal dendrites receive inputs from the entorhinal cortex through the perforant path and from the thalamic projection (Spruston, 2008). The proximal apical dendrite expressed high levels of NR2B-containing NMDAR while distal dendrites expressed lower levels of this receptor (Arrigoni and Greene, 2004). These differences in synaptic inputs and receptor expression control the synaptic integration, transmission, and plasticity of the CA1 pyramidal neuron (Remondes and Schuman, 2002). In this context, TRPM4 in apical dendrites may help to control the local membrane potential and regulate synaptic transmission in a Ca^2+^ -dependent manner (Menigoz et al., 2016), while TRPM4 in the soma may contribute to plateau potential and burst firing. These functions have already been described in several neuronal types (Mrejeru et al., 2011; Kim et al., 2013; Lei et al., 2014). The activation of TRPM4 in CA1 could be involved in developing ADP and repetitive firing during neuronal activity. In this regard, the EPSP or the backpropagated action potential may increase the somatic and dendritic intracellular Ca^2+^ (Larkum et al., 1999), thus boosting TRPM4 activity and increasing the depolarization to a level (plateau potential) sufficient to activate the burst firing mode. Pyramidal neurons in CA1 show different excitability patterns along the septo-temporal axis; ventral hippocampal neurons in CA1 are more excitable and are more depolarized than dorsal CA1 pyramidal neurons (Milior et al., 2016). While we did not explore these differences, we hypothesized that TRPM4 expression is consistent through the axis This is supported by data from Allen brain map showing similar levels in the mRNA through the axis^2^, but further experiments would be necessary to address this point.

Additionally, we found TRPM4 is active at basal non-stimulated conditions, as in smooth muscle cells (Gonzales and Earley, 2012). Consistent with our findings, the resting [Ca^2+^] in pyramidal neurons is between 100 and 150 nM (Wojda et al., 2008), well above the minimum required for TRPM4 activation (Launay et al., 2002, 2004).

### TRPM4-Like Current During Development

During mouse postnatal development, changes in the expression of ion channels and/or receptors determine changes in the morphology and excitability patterns of neurons (Pokorný and Yamamoto, 1981; Johnson-Venkatesh et al., 2015; Sánchez-Aguilera et al., 2020). Our results indicate TRPM4 is expressed at P0, suggesting its expression begins before birth. This is consistent with human evidence that TRPM4 expression in the hippocampus starts at 15 weeks post-conception^3^. At P0, pyramidal neurons display small action potentials and slow activity, which progresses to regular spiking at P14 (Sánchez-Aguilera et al., 2020). While we did not observe a change in the expression of the channel, we observed an increase in the CAN current through postnatal development, corresponding to the changes in firing behavior in CA1 pyramidal neurons from network-driven burst firing to intrinsic burst firing (Wu et al., 2004), but experiments proving the ADP dependency of CAN through development would be required to determine the precise role of this current.

We found that 9-Ph hyperpolarized the membrane potential in neurons at P14 and P35 but not at earlier stages. This change in the membrane potential is accompanied by an increase in input resistance, indicating 9-Ph closes a conductance. These 9-Ph effects are synchronous with an increase in TRPM4-like currents, suggesting that these currents participate in setting the membrane potential. Despite the presence of TRPM4-like currents in early postnatal stages (P0 and P7), we did not observe changes in the membrane potential, suggesting other currents may be shunting the TRPM4-like conductance, including perhaps background potassium conductance (Spigelman et al., 1992), I_*h*_ currents (Vasilyev and Barish, 2002; Bender et al., 2005), or tonic GABA currents (Marchionni et al., 2007). The observation that TRPM4 is expressed at birth strongly suggests TRPM4 expression starts in the prenatal period, in this context, TRPM4 has been implicated in cell migration in epithelial and immune cells (Shimizu et al., 2009; Cáceres et al., 2015), suggesting a similar role in the developing brain; however, TRPM4 KO mice show no differences in volume or number of neurons in the hippocampus compared to wild-type mice (Menigoz et al., 2016), although their connectivity is changed (Bovet-Carmona et al., 2019). Additional experiments using *in utero* electroporation of shRNA against TRPM4 would be necessary to address this point.

In CA1 pyramidal neurons, the localization of TRPM4 in the soma and main apical dendrites allows fine-tuned control of excitability through changes in the membrane potential in a Ca^2+^ dependent manner. Moreover, the increase in the current throughout postnatal development may be a mechanism to control action potential firing during critical periods of neuronal development during the establishment of circuits.

## Data Availability Statement

The raw data supporting the conclusions of this article will be made available by the authors, without undue reservation.

## Ethics Statement

The animal study was reviewed and approved by Ethics Committee, Universidad de Santiago de Chile.

## Author Contributions

DR, OC, and EL-S conducted the experiments. DR and EL-S designed the research and wrote the manuscript. DR, OC, and EL-S edited the manuscript. All authors contributed to the article and approved the submitted version.

## Conflict of Interest

The authors declare that the research was conducted in the absence of any commercial or financial relationships that could be construed as a potential conflict of interest.

## Footnotes

1 https://www.estimationstats.com

2 https://mouse.brain-map.org/gene/show/44509

3 https://www.brainspan.org/lcm/search?exact_match=false&amp;search_term=TRPM4&amp;search_type=gene

## References

[B1] Andersen P. Morris R. Amaral D. Bliss T. O’Keefe J. (2006). *The Hippocampus Book. Illustrated Edition.* Oxford: Oxford University Press.

[B2] Arrigoni E. Greene R. W. (2004). Schaffer collateral and perforant path inputs activate different subtypes of NMDA receptors on the same CA1 pyramidal cell. *Br. J. Pharmacol.* 142 317–322. 10.1038/sj.bjp.0705744 15155538 PMC1574942

[B3] Bender R. A. Galindo R. Mameli M. Gonzalez-Vega R. Valenzuela C. F. Baram T. Z. (2005). Synchronized network activity in developing rat hippocampus involves regional hyperpolarization-activated cyclic nucleotide-gated (HCN) channel function. *Eur. J. Neurosci.* 22 2669–2674. 10.1111/j.1460-9568.2005.04407.x 16307610 PMC2927207

[B4] Bovet-Carmona M. Krautwald K. Menigoz A. Vennekens R. Balschun D. Angenstein F. (2019). Low frequency pulse stimulation of Schaffer collaterals in Trpm4-/- knockout rats differently affects baseline BOLD signals in target regions of the right hippocampus but not BOLD responses at the site of stimulation. *Neuroimage* 188 347–356. 10.1016/j.neuroimage.2018.12.020 30553915

[B5] Bovet-Carmona M. Menigoz A. Pinto S. Tambuyzer T. Krautwald K. Voets T. (2018). Disentangling the role of TRPM4 in hippocampus-dependent plasticity and learning: an electrophysiological, behavioral and FMRI approach. *Brain Struct. Funct.* 223 3557–3576. 10.1007/s00429-018-1706-1 29971514

[B6] Cáceres M. Ortiz L. Recabarren T. Romero A. Colombo A. Leiva-Salcedo E. (2015). TRPM4 is a novel component of the Adhesome required for focal adhesion disassembly, migration and contractility. *PLoS One* 10:e0130540. 10.1371/journal.pone.0130540 26110647 PMC4482413

[B7] Fraser D. D. MacVicar B. A. (1996). Cholinergic-dependent plateau potential in hippocampal CA1 pyramidal neurons. *J. Neurosci.* 16 4113–4128. 10.1523/JNEUROSCI.16-13-04113.1996 8753873 PMC6578995

[B8] Funk G. D. (2013). I think I(CAN): modulation of TRPM4 channels may contribute not only to the emergence of rhythm, but robust output and metabolic sensitivity of the preBötzinger Complex inspiratory network. *J. Physiol.* 591 1593–1594. 10.1113/jphysiol.2012.250811 23547191 PMC3624838

[B9] Gonzales A. L. Earley S. (2012). Endogenous cytosolic Ca2+ buffering is necessary for TRPM4 activity in cerebral artery smooth muscle cells. *Cell Calcium* 51 82–93. 10.1016/j.ceca.2011.11.004 22153976 PMC3265659

[B10] Ho J. Tumkaya T. Aryal S. Choi H. Claridge-Chang A. (2019). Moving beyond P values: data analysis with estimation graphics. *Nat. Methods* 16 565–566. 10.1038/s41592-019-0470-3 31217592

[B11] Jha R. M. Bell J. Citerio G. Hemphill J. C. Kimberly W. T. Narayan R. K. (2020). Role of sulfonylurea receptor 1 and glibenclamide in traumatic brain injury: a review of the evidence. *Int. J. Mol. Sci.* 21:409. 10.3390/ijms21020409 31936452 PMC7013742

[B12] Johnson-Venkatesh E. M. Khan M. N. Murphy G. G. Sutton M. A. Umemori H. (2015). Excitability governs neural development in a hippocampal region-specific manner. *Development* 142 3879–3891. 10.1242/dev.121202 26417041 PMC4712876

[B13] Kim Y. S. Kang E. Makino Y. Park S. Shin J. H. Song H. (2013). Characterizing the conductance underlying depolarization-induced slow current in cerebellar Purkinje cells. *J. Neurophysiol.* 109 1174–1181. 10.1152/jn.01168.2011 23197456 PMC3569132

[B14] Larkum M. E. Zhu J. J. Sakmann B. (1999). A new cellular mechanism for coupling inputs arriving at different cortical layers. *Nature* 398 338–341. 10.1038/18686 10192334

[B15] Launay P. Cheng H. Srivatsan S. Penner R. Fleig A. Kinet J.-P. (2004). TRPM4 regulates calcium oscillations after T cell activation. *Science* 306 1374–1377. 10.1126/science.1098845 15550671

[B16] Launay P. Fleig A. Perraud A.-L. Scharenberg A. M. Penner R. Kinet J.-P. (2002). TRPM4 is a Ca2+-activated nonselective cation channel mediating cell membrane depolarization. *Cell* 109 397–407. 10.1016/s0092-8674(02)00719-5 12015988

[B17] Lei Y.-T. Thuault S. J. Launay P. Margolskee R. F. Kandel E. R. Siegelbaum S. A. (2014). Differential contribution of TRPM4 and TRPM5 nonselective cation channels to the slow afterdepolarization in mouse prefrontal cortex neurons. *Front. Cell. Neurosci.* 8:267. 10.3389/fncel.2014.00267 25237295 PMC4154465

[B18] Leiva-Salcedo E. Riquelme D. Cerda O. Stutzin A. (2017). TRPM4 activation by chemically- and oxygen deprivation-induced ischemia and reperfusion triggers neuronal death. *Channels* 11 624–635. 10.1080/19336950.2017.1375072 28876976 PMC5786181

[B19] Luo Z. Ovcjak A. Wong R. Yang B. Feng Z. Sun H. (2020). Drug development in targeting ion channels for brain edema. *Acta Pharmacol. Sin.* 41 1272–1288. 10.1038/s41401-020-00503-5 32855530 PMC7609292

[B20] Marchionni I. Omrani A. Cherubini E. (2007). In the developing rat hippocampus a tonic GABA A -mediated conductance selectively enhances the glutamatergic drive of principal cells: GABA A -mediated conductance in the developing hippocampus. *J. Physiol.* 581 515–528. 10.1113/jphysiol.2006.125609 17317750 PMC2075167

[B21] Menigoz A. Ahmed T. Sabanov V. Philippaert K. Pinto S. Kerselaers S. (2016). TRPM4-dependent post-synaptic depolarization is essential for the induction of NMDA receptor-dependent LTP in CA1 hippocampal neurons. *Pflugers Arch.* 468 593–607. 10.1007/s00424-015-1764-7 26631168 PMC4792339

[B22] Milior G. Di Castro M. A. Sciarria L. P. Garofalo S. Branchi I. Ragozzino D. (2016). Electrophysiological properties of CA1 pyramidal neurons along the longitudinal axis of the mouse Hippocampus. *Sci. Rep.* 6:38242. 10.1038/srep38242 27922053 PMC5138623

[B23] Mironov S. L. (2008). Metabotropic glutamate receptors activate dendritic calcium waves and TRPM channels which drive rhythmic respiratory patterns in mice. *J. Physiol.* 586 2277–2291. 10.1113/jphysiol.2007.149021 18308826 PMC2479557

[B24] Mrejeru A. Wei A. Ramirez J. M. (2011). Calcium-activated non-selective cation currents are involved in generation of tonic and bursting activity in dopamine neurons of the substantia nigra pars compacta: calcium-activated non-selective cation currents in dopamine neurons. *J. Physiol.* 589 2497–2514. 10.1113/jphysiol.2011.206631 21486760 PMC3115821

[B25] Nilius B. Prenen J. Tang J. Wang C. Owsianik G. Janssens A. (2005). Regulation of the Ca2+ sensitivity of the nonselective cation channel TRPM4. *J. Biol. Chem.* 280 6423–6433. 10.1074/jbc.M411089200 15590641

[B26] O’Malley J. J. Seibt F. Chin J. Beierlein M. (2020). TRPM4 conductances in thalamic reticular nucleus neurons generate persistent firing during slow oscillations. *J. Neurosci.* 40 4813–4823. 10.1523/JNEUROSCI.0324-20.2020 32414784 PMC7326353

[B27] Pedersen S. F. Owsianik G. Nilius B. (2005). TRP channels: an overview. *Cell Calcium* 38 233–252. 10.1016/j.ceca.2005.06.028 16098585

[B28] Picardo M. C. D. Sugimura Y. K. Dorst K. E. Kallurkar P. S. Akins V. T. Ma X. (2019). Trpm4 ion channels in pre-Bötzinger complex interneurons are essential for breathing motor pattern but not rhythm. *PLoS Biol.* 17:e2006094. 10.1371/journal.pbio.2006094 30789900 PMC6400419

[B29] Pokorný J. Yamamoto T. (1981). Postnatal ontogenesis of hippocampal CA1 area in rats. I. Development of dendritic arborisation in pyramidal neurons. *Brain Res. Bull.* 7 113–120. 10.1016/0361-9230(81)90075-7 7272792

[B30] Prawitt D. Monteilh-Zoller M. K. Brixel L. Spangenberg C. Zabel B. Fleig A. (2003). TRPM5 is a transient Ca2+-activated cation channel responding to rapid changes in [Ca2+]i. *Proc. Natl. Acad. Sci. U.S.A.* 100 15166–15171. 10.1073/pnas.2334624100 14634208 PMC299937

[B31] Remondes M. Schuman E. M. (2002). Direct cortical input modulates plasticity and spiking in CA1 pyramidal neurons. *Nature* 416 736–740. 10.1038/416736a 11961555

[B32] Riquelme D. Silva I. Philp A. M. Huidobro-Toro J. P. Cerda O. Trimmer J. S. (2018). Subcellular localization and activity of TRPM4 in medial prefrontal cortex layer 2/3. *Front. Cell. Neurosci.* 12:12. 10.3389/fncel.2018.00012 29440991 PMC5797675

[B33] Robert S. M. Reeves B. C. Alper S. L. Zhang J. Kahle K. T. (2020). New drugs on the horizon for cerebral edema: What’s in the clinical development pipeline? *Expert Opin. Investig. Drugs* 29 1099–1105. 10.1080/13543784.2020.1813715 32815401 PMC8104020

[B34] Sánchez-Aguilera A. Monedero G. Colino A. Vicente-Torres M. Á. (2020). Development of action potential waveform in hippocampal CA1 pyramidal neurons. *Neuroscience* 442 151–167. 10.1016/j.neuroscience.2020.06.042 32634531

[B35] Schattling B. Steinbach K. Thies E. Kruse M. Menigoz A. Ufer F. (2012). TRPM4 cation channel mediates axonal and neuronal degeneration in experimental autoimmune encephalomyelitis and multiple sclerosis. *Nat. Med.* 18 1805–1811. 10.1038/nm.3015 23160238

[B36] Shimizu T. Owsianik G. Freichel M. Flockerzi V. Nilius B. Vennekens R. (2009). TRPM4 regulates migration of mast cells in mice. *Cell Calcium* 45 226–232. 10.1016/j.ceca.2008.10.005 19046767

[B37] Shin J. H. Kim Y. S. Worley P. F. Linden D. J. (2009). Depolarization-induced slow current in cerebellar Purkinje cells does not require metabotropic glutamate receptor 1. *Neuroscience* 162 688–693. 10.1016/j.neuroscience.2009.01.044 19409231 PMC2716407

[B38] Shpak G. Zylbertal A. Yarom Y. Wagner S. (2012). Calcium-activated sustained firing responses distinguish accessory from main olfactory bulb Mitral Cells. *J. Neurosci.* 32 6251–6262. 10.1523/jneurosci.4397-11.2012 22553031 PMC6622135

[B39] Soriano E. Del Río J. A. Martínez A. Supèr H. (1994). Organization of the embryonic and early postnatal murine hippocampus. I. Immunocytochemical characterization of neuronal populations in the subplate and marginal zone. *J. Comp. Neurol.* 342 571–595. 10.1002/cne.903420406 7913715

[B40] Spigelman I. Zhang L. Carlen P. L. (1992). Patch-clamp study of postnatal development of CA1 neurons in rat hippocampal slices: membrane excitability and K+ currents. *J. Neurophysiol.* 68 55–69. 10.1152/jn.1992.68.1.55 1517828

[B41] Spruston N. (2008). Pyramidal neurons: dendritic structure and synaptic integration. *Nat. Rev. Neurosci.* 9 206–221. 10.1038/nrn2286 18270515

[B42] Supèr H. Soriano E. (1994). The organization of the embryonic and early postnatal murine hippocampus. II. Development of entorhinal, commissural, and septal connections studied with the lipophilic tracer DiI. *J. Comp. Neurol.* 344 101–120. 10.1002/cne.903440108 8063952

[B43] Teruyama R. Sakuraba M. Kurotaki H. Armstrong W. E. (2011). Transient receptor potential channel m4 and m5 in magnocellular cells in rat supraoptic and paraventricular nuclei. *J. Neuroendocrinol.* 23 1204–1213. 10.1111/j.1365-2826.2011.02211.x 21848647 PMC5703211

[B44] Trimmer J. S. (2015). Subcellular Localization of K+ channels in mammalian brain neurons: remarkable precision in the midst of extraordinary complexity. *Neuron* 85 238–256. 10.1016/j.neuron.2014.12.042 25611506 PMC4303806

[B45] Vasilyev D. V. Barish M. E. (2002). Postnatal development of the hyperpolarization-activated excitatory current Ih in mouse hippocampal pyramidal neurons. *J. Neurosci.* 22 8992–9004. 10.1113/jphysiol.2004.069104 12388606 PMC6757670

[B46] Wojda U. Salinska E. Kuznicki J. (2008). Calcium ions in neuronal degeneration. *IUBMB Life* 60 575–590. 10.1002/iub.91 18478527

[B47] Wu W. W. Chan C. S. Disterhoft J. F. (2004). Slow Afterhyperpolarization governs the development of NMDA Receptor–dependent afterdepolarization in CA1 pyramidal neurons during synaptic stimulation. *J. Neurophysiol.* 92 2346–2356. 10.1152/jn.00977.2003 15190096

