# Supplementary material for: TRPM4 Expression During Postnatal Developmental of Mouse CA1 Pyramidal Neurons
Source: Front Neuroanat. 2021 Apr 28;15:643287. doi: 10.3389/fnana.2021.643287 (PMC8113704; doi:10.3389/fnana.2021.643287)
Supplement: Supplementary file 1 [file Table_1.pdf]

Supplementary Table 1

Table 1
List of antibodies

Antibody	Isotype	Dilution	Final [ ], µg/mL	Source	Type	Catalog#	RRDI	Purification
Mouse anti-TRPM4	IgM	NDS		Neuromab	M	73-501	AB_2716758	none
Mouse anti-Ankyrin G	IgG2a	1:100	8	Neuromab	M	75-146	AB_10673030	AP
Rabbit anti-MAP2	IgG	1:300	3	Abcam	P	ab32454	AB_776174	AP
Alexa Fluor 488 conjugated goat anti-mouse IgM	IgG	1:2000	1	ThermoFisher Scientific	P	A21042	AB_2535711	AP
Alexa Fluor 546 conjugated goat anti-mouse IgG2a	IgG	1:2000	1	ThermoFisher Scientific	P	A21133	AB_2535772	AP
Alexa Fluor 546 conjugated donkey anti-rabbit IgG	IgG	1:2000	1	ThermoFisher Scientific	P	A10040	AB_2534016	AP

NDS: Non-diluted supernatant
M: Monoclonal
P: Polyclonal
AP: Affinity purified
